# Engineering Acetogenic Bacteria for Efficient One-Carbon Utilization

**DOI:** 10.3389/fmicb.2022.865168

**Published:** 2022-05-09

**Authors:** Hyeonsik Lee, Jiyun Bae, Sangrak Jin, Seulgi Kang, Byung-Kwan Cho

**Affiliations:** ^1^Department of Biological Sciences, Korea Advanced Institute of Science and Technology, Daejeon, South Korea; ^2^KI for the BioCentury, Korea Advanced Institute of Science and Technology, Daejeon, South Korea

**Keywords:** acetogenic bacteria, one-carbon utilization, Wood–Ljungdahl pathway, energy metabolism, biocatalyst

## Abstract

C1 gases, including carbon dioxide (CO_2_) and carbon monoxide (CO), are major contributors to climate crisis. Numerous studies have been conducted to fix and recycle C1 gases in order to solve this problem. Among them, the use of microorganisms as biocatalysts to convert C1 gases to value-added chemicals is a promising solution. Acetogenic bacteria (acetogens) have received attention as high-potential biocatalysts owing to their conserved Wood–Ljungdahl (WL) pathway, which fixes not only CO_2_ but also CO. Although some metabolites have been produced *via* C1 gas fermentation on an industrial scale, the conversion of C1 gases to produce various biochemicals by engineering acetogens has been limited. The energy limitation of acetogens is one of the challenges to overcome, as their metabolism operates at a thermodynamic limit, and the low solubility of gaseous substrates results in a limited supply of cellular energy. This review provides strategies for developing efficient platform strains for C1 gas conversion, focusing on engineering the WL pathway. Supplying liquid C1 substrates, which can be obtained from CO_2_, or electricity is introduced as a strategy to overcome the energy limitation. Future prospective approaches on engineering acetogens based on systems and synthetic biology approaches are also discussed.

## Introduction

The rapid increase in fossil fuel usage and greenhouse gas emissions has caused one of the biggest problems for humankind today. C1 gases such as carbon dioxide (CO_2_) and carbon monoxide (CO), which constitute greenhouse gases, industrial waste gases, and synthesis gases (syngas), are the main culprits of the climate crisis (Anwar et al., 2018; Ritchie and Roser, 2020). To make the earth a sustainable place, reducing emissions is crucial, and urgent solutions for carbon capturing, utilization, and storage are needed.

C1 gas fermentation could be a solution, which utilizes microbes as biocatalysts. This is a preferable approach, because it does not require high pressure, temperature, cost, and energy, unlike chemical catalysts, such as in the Fischer–Tropsch process (Latif et al., 2014; Dürre, 2017; Köpke and Simpson, 2020). C1 gases are utilized by microbes as feedstocks and finally converted to value-added chemicals under mild conditions that are required for the optimal growth of microbes.

Acetogenic bacteria (acetogens) are promising platform microbes for C1 gas fixation. They are facultative autotrophs that fix CO_2_ and CO as carbon or energy sources *via* the unique metabolic pathway, the Wood–Ljungdahl (WL) pathway (Ragsdale and Pierce, 2008; Drake et al., 2013). Of the CO_2_-fixing pathways known to date, the WL pathway is considered the most energetically efficient (Fast et al., 2015; Claassens et al., 2019). In addition, it is the only pathway for CO_2_ fixation coupled with an energy conservation system that plays a crucial role in generating cellular energy and sustaining life (Schuchmann and Müller, 2014). Numerous studies have utilized acetogens as biocatalysts to convert C1 gases into value-added chemicals (Berzin et al., 2012, 2013; Köpke et al., 2012; Banerjee et al., 2014; Beck et al., 2014; Woolston et al., 2018; Annan et al., 2019; Huang et al., 2019; Jin et al., 2020; Bae et al., 2022; Liew et al., 2022). Among the native metabolites produced from acetogens, acetate, ethanol, and 2,3-butanediol (2,3-BDO) have been produced by C1 gas fermentation on an industrial scale using a non-engineered strain of *Clostridium autoethanogenum* (Marcellin et al., 2016). Although numerous efforts have been made to engineer acetogens to produce various biochemicals from C1 gases, these studies have been limited to a small-scale. The low energy potential of C1 gases compared to that of glucose is one of the limitations that causes slow growth and low productivity under autotrophic conditions. In addition, acetogenesis overall has a small change in free energy that results in thermodynamic constraints and the synthesis of less than one molecule ATP (Schuchmann and Müller, 2014). The energy limitations of acetogens and the low solubility of gaseous substrates are additional hurdles to overcome, as they can ultimately limit the availability of cellular energy, thereby restricting the production of value-added metabolites (Abubackar et al., 2011; Molitor et al., 2017). Therefore, it is necessary to engineer the acetogenic metabolism, including the WL pathway and energy metabolism, and alleviate the solubility issue by supplying liquid C1 substrates (e.g., methanol and formate) or electricity as alternative electron sources. This will enable to fully exploit the potential of acetogens as biocatalysts for C1 utilization.

In this review, the physiology and metabolism of acetogens are addressed, focusing on the WL pathway and the energy conservation system. Strategies to enhance C1 gas fixation efficiency by engineering the WL pathway and overcoming energy limitations under autotrophic conditions are also introduced. Furthermore, future perspectives on engineering acetogens to achieve highly efficient biocatalysts are discussed.

## Understanding Physiology and Metabolism of Acetogens

To date, over 100 acetogens belonging to 23 genera have been isolated that can grow in diverse environments such as under a wide range of temperature and pH. Depending on the species, various biochemicals, including acetate, ethanol, butyrate, or 2,3-BDO, can be produced from C1 feedstocks (Table 1). As an essential precursor for synthesizing these products, acetyl-CoA is generated through the WL pathway in acetogens from either CO_2_ or CO. As CO_2_ can only serve as a carbon source, CO_2_ fixation requires an additional source of energy such as H_2_ or CO. Although CO can serve as both carbon and energy sources, the addition of H_2_ is desirable to refix CO_2_ generated from CO oxidation, as two-thirds of the carbon is lost when CO is used as a sole substrate (Jeong et al., 2015; Bertsch and Müller, 2015b; Weghoff et al., 2016). Along with the WL pathway, the energy metabolism in acetogens also generates reducing equivalents from H_2_ and CO. Accordingly, the composition of gas mixtures fed in acetogens affects not only the carbon yield of the bioprocess but also the metabolism of acetogens, which points out that understanding the acetogenic metabolism is important to realize the efficient C1 gas fermentation.

**Table 1:** Features of diverse acetogenic species.

Organism	Substrate	Products	Optimal growth temperature (°C)	Optimal pH	Genome complete levels	References
*Acetobacterium bakii*	H_2_/CO_2_, CO, methanol	Acetate	20	6.5	Scaffold	Kotsyurbenko et al., 1995; Hwang et al., 2015
*Acetobacterium woodii*	H_2_/CO_2_, methanol, formate	Acetate	30	7.0	Complete	Balch et al., 1977; Bache and Pfennig, 1981; Poehlein et al., 2012
*Acetohalobium arabaticum*	H_2_/CO_2_, CO	Acetate	38–40	7.6–8.0	Complete	Zhilina and Zavarzin, 1990; Sikorski et al., 2010
*Blautia producta*	H2/CO2, CO	Acetate	37	7.0	Complete	Lorowitz and Bryant, 1984; Geerligs et al., 1987; Liu et al., 2008; Tourlousse et al., 2020
*Clostridium aceticum*	H_2_/CO_2_, CO	Acetate	30	8.3	Complete	Wieringa, 1936; Braun et al., 1981; Lux and Drake, 1992; Poehlein et al., 2015b
*Clostridium autoethanogenum*	H_2_/CO_2_, CO	2,3-BDO, acetate, ethanol	37	6.0	Complete	Abrini et al., 1994; Köpke et al., 2011; Brown et al., 2014
*Clostridium carboxidivorans*	H_2_/CO_2_, CO	Acetate, ethanol, butyrate, butanol	38	5.0–7.0	Complete	Liou et al., 2005; Li et al., 2015
*Clostridium coskatii*	H_2_/CO_2_, CO	Acetate, ethanol	37	6.0	Contig	Zahn and Saxena, 2012; Bengelsdorf et al., 2016
*Clostridium drakei*	H_2_/CO_2_, CO	Acetate, ethanol, butyrate	30	5.4–7.5	Complete	Küsel et al., 2000; Liou et al., 2005; Gossner et al., 2008; Jeong et al., 2014
*Clostridium formicaceticum*	CO, CH_3_OH	Acetate, formate	37	8.1	Complete	Andreesen et al., 1970; Lux and Drake, 1992; Karl et al., 2017
*Clostridium ljungdahlii*	H_2_/CO_2_, CO, formate	2,3-BDO, acetate, ethanol	37	6.0	Complete	Tanner et al., 1993; Köpke et al., 2010, 2011
*Clostridium magnum*	H_2_/CO_2_, methanol	Acetate	30	7.2	Scaffold	Schink, 1984; Bomar et al., 1991; Uhlig et al., 2016
*Clostridium ragsdalei*	H_2_/CO_2_, CO	2,3-BDO, acetate, ethanol	37	6.3	Contig	Huhnke et al., 2008; Köpke et al., 2011; Bengelsdorf et al., 2016
*Clostridium scatologenes*	H_2_/CO_2_, CO, formate	Acetate, ethanol, butyrate	37	5.4–7.0	Complete	Liou et al., 2005; Zhu et al., 2015
*Eubacterium limosum*	H_2_/CO_2_, CO, methanol, formate	Acetate, Butyrate	37	7.0	Complete	Eggerth, 1935; Genthner et al., 1981; Genthner and Bryant, 1982; Genthner and Bryant, 1987; Song and Cho, 2015
*Eubacterium callanderi*	H_2_/CO_2_, CO, methanol	Acetate, butyrate	37	7.0	Complete	Chang et al., 1997, 2001; Roh et al., 2011
*Sporomusa ovata*	H_2_/CO_2_, methanol, formate	Acetate	34	6.3	Scaffold	Möller et al., 1984; Poehlein et al., 2013
*Thermoacetogenium phaeum*	H_2_/CO_2_, methanol, formate	Acetate	60	6.8	Complete	Hattori et al., 2000; Oehler et al., 2012
*Thermoanaerobacter kivui*	H_2_/CO_2_, CO, formate	Acetate	66	6.4	Complete	Leigh et al., 1981; Daniel et al., 1990; Hess et al., 2014
*Treponema primitia*	H_2_/CO_2_	Acetate	30	7.2	Complete	Graber et al., 2004; Graber and Breznak, 2004; Rosenthal et al., 2011
*Moorella thermoacetica*	H_2_/CO_2_, CO, methanol, formate	Acetate	55	7.0	Complete	Fontaine et al., 1942; Kerby and Zeikus, 1983; Daniel et al., 1990; Bengelsdorf et al., 2015; Poehlein et al., 2015a

### Wood–Ljungdahl Pathway

**Figure 1:**
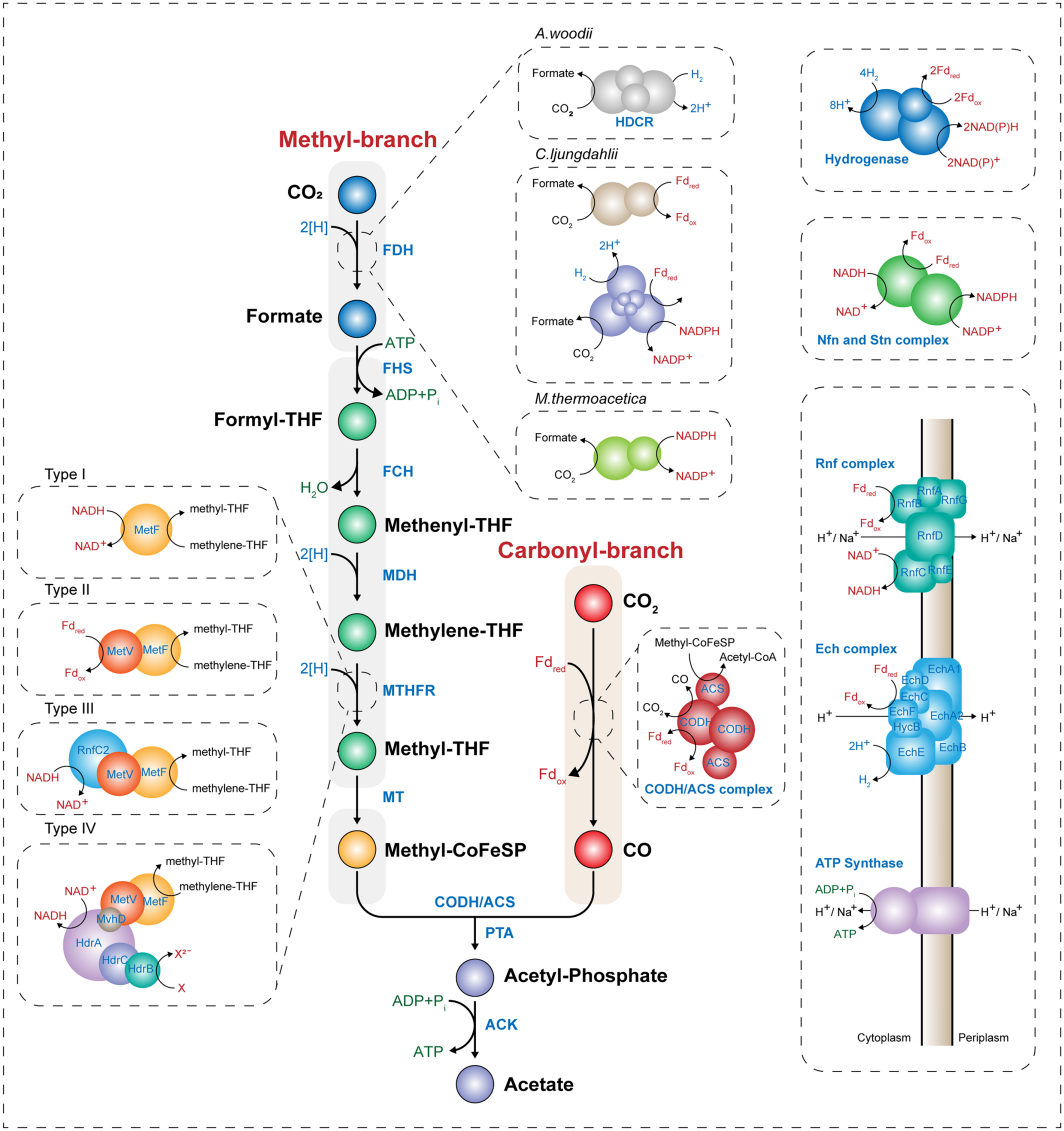
Scheme of the Wood–Ljungdahl pathway and energy conservation. The Wood–Ljungdahl pathway and energy conservation system in acetogens. CO, carbon monoxide; CO_2_, carbon dioxide; THF, tetrahydrofolate; FDH, formate dehydrogenase; FHS, formyl-tetrahydrofolate synthase; FCH, formyl-cyclohydrolase; MDH, methylene-tetrahydrofolate dehydrogenase; MTHFR, methylene-tetrahydrofolate reductase; MT, methyltransferase; CoFeSP, corrinoid iron–sulfur protein; CODH, CO dehydrogenase; ACS, acetyl-CoA synthase; PTA, phosphotransacetylase; ACK, acetate kinase; HDCR, hydrogen-dependent CO_2_ reductase; Fd_ox_, oxidized ferredoxin; Fd_red_, reduced ferredoxin; and P_i_, inorganic phosphate.

The WL pathway is mainly composed of two linear metabolic branches: a methyl- and a carbonyl-branch (Figure 1). The methyl-branch consists of six reactions, starting with the reduction of CO2 to formate using two reducing equivalents, and formate is transferred to tetrahydrofolate (THF) to generate formyl-THF, consuming one molecule of ATP. Water splits from formyl-THF to generate methenyl-THF, which is further reduced *via* methylene-THF to methyl-THF. Finally, the methyl group is transferred to acetyl-CoA synthase (ACS) *via* the corrinoid iron–sulfur protein (CoFeSP). Most genes involved in the methyl-branch are strongly conserved among phylogenetically diverse acetogens. However, some genes have diverse characteristics depending on the species, such as the formation of various protein complexes or the use of different cofactors (Shin et al., 2016). Unlike the methyl-branch, the carbonyl-branch undergoes a one-step reaction by a multi-component enzyme called carbon monoxide dehydrogenase/acetyl-CoA synthase (CODH/ACS), which synthesizes acetyl-CoA by attaching a carbonyl-group from the reduction of CO_2_, or CO directly, to methyl-CoFeSP generated from the methyl-branch. Acetyl-CoA can be used as a building block for various chemicals. Consequently, C1 gas fixation using the WL pathway requires only one molecule of ATP, which is the lowest energy requirement among all existing biological CO_2_ fixing metabolic pathways (Fast and Papoutsakis, 2012). Thus, acetogens have received attention as biocatalysts that can efficiently fix C1 gases. In this section, we compare and summarize the processes of C1 assimilation by several major acetogens.

#### Methyl-Branch

The methyl-branch is a linear metabolic branch that converts CO_2_ to methyl groups. To compare the methyl-branch between the acetogenic species, we discuss the reactions of the methyl-branch into the following three parts: (i) the first step in which CO_2_ is converted to formate, (ii) the second step in which formate is converted to methyl-THF, and (iii) the last step in which methyl-THF is converted to methyl-CoFeSP (Figure 1).

##### Step 1: CO_2_ to Formate

The first step in the methyl-branch is the reduction of CO_2_ to formate by selenocysteine- or non-selenocysteine-containing formate dehydrogenase (FDH). As the standard redox potential of this reaction is −432 mV, NADH (−320 mV) is not sufficient as an electron donor to drive this reaction. Instead, ferredoxin (−450 to −500 mV), H_2_ (−414 mV), and NADPH (−370 mV) can be used for the reduction of CO_2_ to formate (Wang et al., 2013; Schuchmann and Müller, 2014). Depending on the electron delivery system, model acetogens are divided into three types. The first model acetogen, *Acetobacterium woodii*, uses hydrogen-dependent CO_2_ reductase (HDCR), an FDH complex linked to hydrogenase, to receive electrons directly from hydrogen and convert CO_2_ to formate. In the case of the second model species, *Clostridium ljungdahlii*, reduced ferredoxin (Fd_red_) or NADPH is used as cofactor for the two types of FDH. The third model acetogen, *Moorella thermoacetica*, converts CO_2_ into formate using only NADPH as a cofactor (Figure 1; Wang et al., 2013; Schuchmann and Müller, 2014; Shin et al., 2016).

##### Step 2: Formate to Methyl-THF

The second step is the conversion of the formyl-group to the methyl group in THF. In this process, one molecule of ATP and four electrons are consumed. First, formyl-THF synthetase (FHS) uses one ATP molecule to bind formate to THF to form formyl-THF. Subsequently, methyl-THF cyclohydrolase (FCH) cyclizes the formyl-group linked to THF to generate a methenyl intermediate, and methylene-THF is finally converted by methylene-THF dehydrogenase (MDH). In *M. thermoacetica*, cyclohydrolase and dehydrogenase function as a single bifunctional protein complex, whereas *Clostridium formicoaceticum* (Clark et al., 1982) and *A. woodii* (Ragsdale and Ljungdahl, 1984) have monofunctional proteins. In particular, when converting methenyl-THF to methylene-THF, two electrons are used and transferred by either NADH or NADPH depending on the species (Moore et al., 1974; Ragsdale and Ljungdahl, 1984). The last step is the conversion of methylene-THF to methyl-THF by methylene-THF reductase (MTHFR). Interestingly, this enzyme reaction is classified into four types depending on the acetogenic species (Öppinger et al., 2022). In Type I, MetF alone generates methyl-THF using one molecule of NADH in *E. coli* (Sheppard et al., 1999), *Thermus thermophilus* (Igari et al., 2011), and *Blautia producta* (Wohlfarth et al., 1990). The Type II MTHFR system consists of the MetV-MetF complex and is found in *C. formicoaceticum*, *C. ljungdahlii*, and *Thermoanaerobacter kivui*. This Type II MTHFR complex protein obtains electrons from Fd_red_ to convert methylene-THF to methyl-THF (Dietrich et al., 2021; Katsyv et al., 2021; Wiechmann and Müller, 2021). The Type III system used by *A. woodii* has MetV-MetF bound to RnfC2, which has a stoichiometry of 1:1:1, and NADH is used as a cofactor (Bertsch et al., 2015). Finally, in the Type IV MTHFR system, MetV-MetF constitutes a complex in which HdrCBA and MvhD are connected. This complex may use electron bifurcation using two molecules of NADH cofactor to generate methyl-THF and one molecule of a reduced electron carrier (e.g., Fd_red_). Among acetogens, *M. thermoacetica* and *Sporomusa ovata* are known to have the Type IV MTHFR system (Figure 1; Mock et al., 2014). Such differences appear to be due to the utilization of various C1 substrates, including CO and methanol, or difference in optimal growth conditions (e.g., temperature, pH, or metal cofactor), and play a role in the optimal regulation of the intracellular redox balance.

##### Step 3: Methyl-THF to Methyl-CoFeSP

The final step is to form methyl-CoFeSP by transferring methyl groups from methyl-THF to CoFeSP by methyltransferase (MT; Figure 1). THF, from which the methyl group has been removed, is recycled by several metabolic reactions. Methyl-CoFeSP, the final product of the methyl-branch of the WL pathway, is converted into Acetyl-CoA while transferring the methyl group to the CODH/ACS complex of the carbonyl-branch, and the CoFeSP is also recycled.

#### Carbonyl-Branch

The carbonyl-branch is a pathway for synthesizing acetyl-CoA by combining a carbonyl-group with methyl-CoFeSP obtained from the methyl-branch. When CO is a substrate, CO can be directly incorporated into the carbonyl-branch, while CO_2_ can be used after its reduction to CO. These reactions are catalyzed by the CODH/ACS complex. CODH catalyzes either the reduction of CO_2_ to CO or the oxidation of CO to CO_2_ (Figure 1). It is a homodimeric enzyme that contains five Fe-S clusters. Because the CO_2_ to CO reduction reaction is the largest thermodynamic barrier in the WL pathway and has a very low standard redox potential (*E*_0_’ = −520 mV; Thauer et al., 1977), CODH enzymes in most acetogens use only Fd_red_ as an electron donor. Some acetogenic species such as *M. thermoacetica* and *C. ljungdahlii*, which are capable of CO oxidation, have a Ni-Fe-S reaction center in the C-cluster, and Ni insertion accessory proteins are used to construct the Ni insertion C-cluster. Hence, these protein families play an essential role in the growth of the acetogen on CO under autotrophic conditions. When the gene encoding the Ni insertion accessory protein, such as *cooC*, is deleted, the mutant strain needs a high Ni cation concentration for autotrophic growth on CO (Kerby et al., 1997; Jeon et al., 2001).

ACS is a protein containing an A-cluster with a Ni–Ni-Fe_4_S_4_ active site. This enzyme catalyzes the synthesis of acetyl-CoA, combining CO with methyl-CoFeSP obtained from the carbonyl- and methyl-branch, respectively. Protein crystal analysis of the CODH/ACS complex showed that this complex has two structural forms. In the closed-form case, a gas tunnel is generated from the C-cluster of CODH to the A-cluster of the ACS protein. Along this tunnel, one molecule of CO can be transferred from the C-cluster to the A-cluster, and carboxylation of CO with methyl-CoFeSP in the A-cluster results in acetyl-CoA production. Next, in the open-form, the CO gas tunnel is disconnected by a conformational change in the CODH/ACS complex. In this form, methylation of the A-cluster becomes possible as CoFeSP can access to the active site of A-cluster (Doukov et al., 2008). Acetyl-CoA can be synthesized through this conformational change by attaching a CO molecule and a CoA cofactor to methyl-CoFeSP in the CODH/ACS complex.

Through the cooperation of the methyl- and carbonyl-branch, acetogens synthesize one molecule of acetyl-CoA from two molecules of CO_2_ or CO. Acetyl-CoA is an intracellular building block in living organisms and is used to increase cell mass or synthesize one molecule of acetate through SLP *via* catalytic reactions of phosphotransacetylase (PTA) and acetate kinase (ACK). Therefore, the WL pathway consumes one molecule of ATP to convert formate to formyl-THF and produces one molecule of ATP through SLP during acetate synthesis, resulting in a net ATP yield of zero.

### Energy Metabolism

As one molecule of ATP is consumed for fixation of C1 gases through the WL pathway, additional cellular energy is required for acetogens to grow C1 gas conditions. Although the oxidation of CO generates Fd_red_, acetogens usually require an additional energy source, such as H_2_, to obtain reducing equivalents and to fix the residual CO_2_ produced from the oxidation of CO. Hydrogenase has diverse protein characteristics in different microbial species but the common role is to generate Fd_red_ or NAD(P)H from H_2_. Electron bifurcation found in acetogens is a mechanism of biological energy conservation that couples the exergonic oxidation and endergonic reduction reactions. (Peters et al., 2016). Acetogens have electron-bifurcating hydrogenases that couple the exergonic reduction of NAD^+^ to endergonic reduction of ferredoxin with exergonic oxidation of H_2_ by making the overall reaction exergonic (Figure 1; Schuchmann and Müller, 2014). During C1 gas fixation, reducing equivalents are supplied *via* electron carriers to the redox reactions involved in the WL or other metabolic pathways for cell growth. Interestingly, Fd_red_ produced by CODH or hydrogenase generates an ion gradient between cellular membranes *via* the membrane-bound respiratory enzyme complex of acetogens and yields additional ATP using membrane-bound ATP synthase. Generation of ATP *via* these respiratory enzyme complexes involved in energy conservation systems is the mode used by acetogens to sustain their lives.

There are two different complexes used by acetogens, the Rnf and Ech complexes (Figure 1). The Rnf complex is composed of six subunits, as found in *A. woodii*, *C. ljungdahlii*, and *C. autoethanogenum*, and has a ferredoxin:NAD^+^ oxidoreductase activity (Biegel and Müller, 2010). It receives electrons from Fd_red_ and transfers them to NAD^+^ to generate NADH. It pumps cations such as H^+^ or Na^+^ from inside the cells. In contrast, the Ech complex, which consists of eight or nine subunits and is found in *M. thermoacetica* and *T. kivui*, has ferredoxin:H^+^ oxidoreductase activity that uses protons instead of NAD^+^ as the final electron acceptor and pumps out protons_,_ during the generation of hydrogen (Hedderich and Forzi, 2005). The generated electrochemical ion gradient results in ATP synthesis by the membrane-bound ATP synthase. The Rnf-containing, Na^+^-dependent acetogen *A. woodii* is known to produce one molecule of ATP per 3.3 Na^+^, as experimentally determined by solving the crystal structure of *A. woodii* ATP synthase (Matthies et al., 2014). In contrast, a one molecule yield of ATP per 4 H^+^ is assumed for other species such as *C. ljungdahlii* and *M. thermoacetica* (Schuchmann and Müller, 2014).

In addition, some acetogens such as *C. autoethanogenum* and *S. ovata* have been reported to have NADH-dependent reduced ferredoxin:NADP^+^ oxidoreductase (Nfn) and *Sporomusa*-type Nfn (Stn), respectively (Figure 1; Wang et al., 2013; Kremp et al., 2020; Mahamkali et al., 2020). The Nfn complex is composed of two subunits of NfnAB, each of which is known to have several Fe-S reaction centers. It is known for its redox balancing potential by producing NADPH from Fd_red_ and NADH through electron bifurcation (Mahamkali et al., 2020). Therefore, both energy conservation and redox balancing systems are highly important for acetogens to generate cellular energy during autotrophic growth under C1 gas conditions.

## Engineering the WL Pathway to Enhance the Efficiency of C1 Gas Fixation

Because C1 gases, unlike other substrates such as glucose or glycerol, are gaseous substrates, the gas-to-liquid mass transfer rate is critically affected by the physical properties of gas solubility. Many studies have attempted to increase the fixing efficiency of C1 gases through various gas fermentation techniques, such as increasing the partial pressure of the gas (Phillips et al., 1993; Bredwell and Worden, 1998; Hurst and Lewis, 2010; Orgill et al., 2013; Sathish et al., 2019; Bae et al., 2022). However, this approach also has physical limitations, and thus, genetic attempts should be made to overcome the low productivity, yield, and cell density of acetogens by developing a platform acetogen strain with increased C1 gas fixation efficiency and expanding it to a commercial scale. This chapter summarizes a few approaches that use simple genetic manipulation to increase C1 gas fixation efficiency of acetogens.

### Engineering of the Methyl-Branch in the WL Pathway

**Figure 2:**
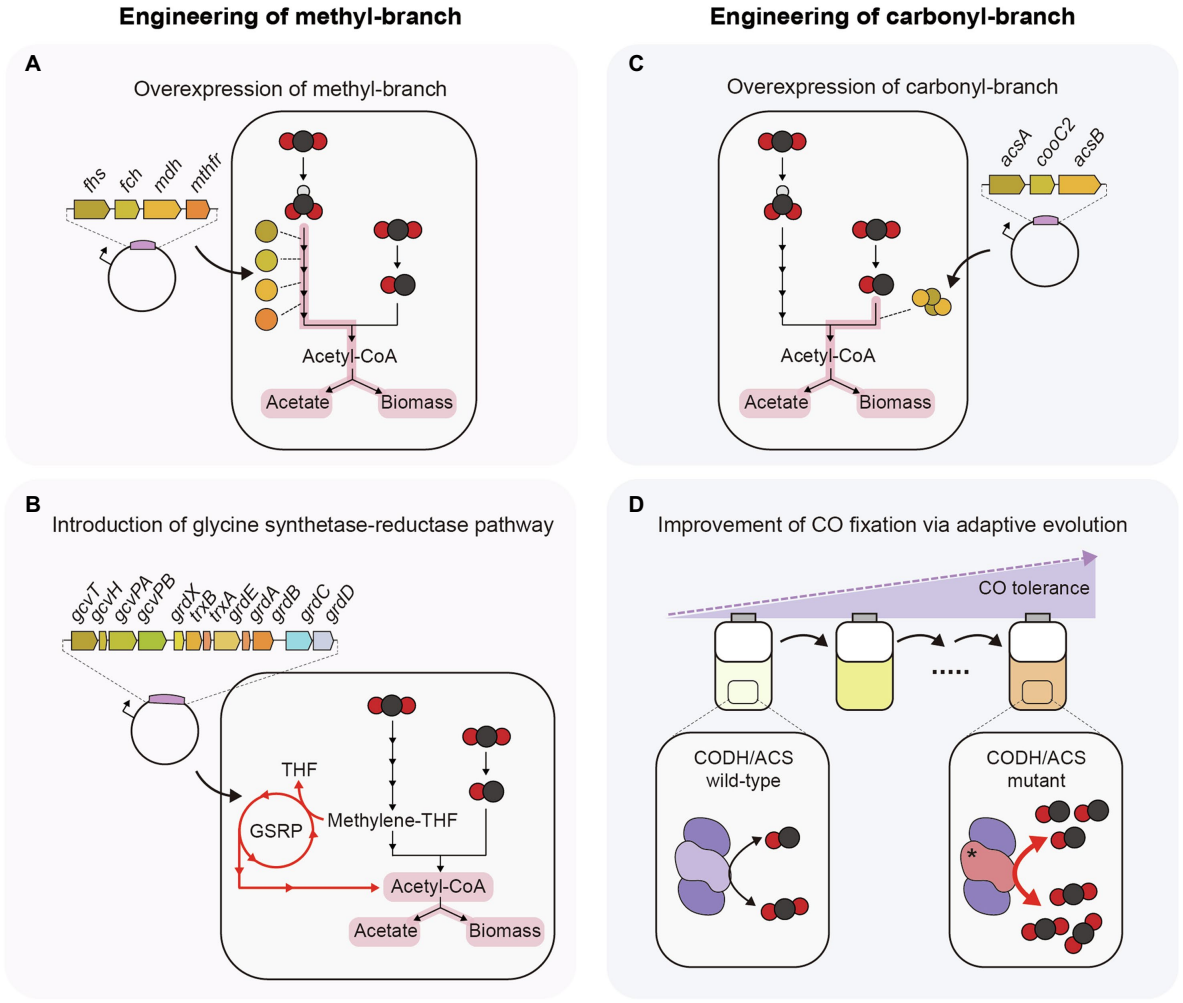
Strategies to enhance efficiency of C1 gas fixation in acetogens. **(A)** Plasmid-based overexpression of THF-dependent enzymes. **(B)** Introduction of glycine synthase-reductase pathway. **(C)** Plasmid-based overexpression of CODH, ACS, and CooC2. **(D)** CODH/ACS mutant obtained from adaptive laboratory evolution under a high concentration of CO. GSRP, Glycine synthase-reductase pathway.

To increase the C1 gas fixation efficiency *via* the WL pathway, the most straightforward approach is to overexpress genes encoding enzymes of the methyl-branch (Figure 2A). When four THF-dependent enzymes (FHS, FCH, MDH, and MTHFR) of *C. ljungdahlii* were overexpressed in *A. woodii*, its growth rate increased approximately 1.1-fold and acetate production increased approximately 1.2-fold compared to the empty vector control under C1 autotrophic batch cultivation. In addition, the engineered strain showed approximately a 1.6-fold increase in the specific activity of ACK, whereas strains overexpressing PTA or ACK showed approximately a 1.2-fold increase in specific activity of ACK (Straub et al., 2014). This result indicated that CO_2_ fixation efficiency can be increased by upregulating the genes involved in the methyl-branch.

Another way to increase CO_2_ fixation efficiency is to introduce a new metabolic pathway that can fix CO_2_ in connection with the WL pathway (Figure 2B). An example is the glycine synthase-reductase pathway (GSRP). *Clostridium drakei* has a unique CO_2_-fixing metabolic pathway, in which the WL pathway and GSRP are connected. Methylene-THF, an intermediate of the methyl-branch of the WL pathway, is converted to glycine or serine through GSRP or the reductive glycine pathway (RGP), and these amino acids are converted to acetate through acetyl-phosphate. In this process, one ATP molecule can be synthesized through SLP. Heterologous expression of GSRP in *Eubacterium limosum* showed that the CO_2_ consumption rate and acetate production rate increased 1.5-fold (Song et al., 2020). These results suggest the possibility of increasing the C1 gas-fixing efficiency by introducing an additional C1 gas-fixing metabolic pathway that can be connected to the WL pathway.

### Engineering of the Carbonyl-Branch in the WL Pathway

As mentioned above, the only way to utilize CO as carbon and energy sources in acetogens is using the carbonyl-branch of the WL pathway, and CODH/ACS complex is involved in this process. *C. autoethanogenum* and *T. kivui* can grow well under CO autotrophic condition (Weghoff et al., 2016; Liew et al., 2017), whereas some acetogens such as *A. woodii* and *E. limosum* show low CO oxidation rate and growth retardation at a high concentration of CO (Bertsch and Müller, 2015b; Kang et al., 2020). Hence, engineering the CODH/ACS complex is necessary for efficient utilization of CO and improving growth on CO in these acetogens. To increase the CO oxidation rate, the most straightforward approach is to overexpress all genes encoding proteins of the CODH/ACS complex (Figure 2C). The overexpression of *acsA*-encoding CODH, *acsB* encoding ACS, and *cooC2* encoding maturation protein in *Eubacterium callanderi* KIST612 using plasmids led to an increase in the CO oxidation and acetate production rates by 3.1-fold and 1.4-fold, respectively, compared to the control strain, whereas there was no difference when individual genes were overexpressed (Kang et al., 2021).

In one case, the CO oxidation rate was increased by changing the protein sequence of CODH/ACS but not by overexpression of the CODH/ACS complex (Figure 2D). In this study, adaptive laboratory evolution was performed on the *E. limosum* ATCC8486 strain under 44% CO conditions to enhance tolerance to CO. In the evolved strain, a C290A single-nucleotide variation (SNV) was found in the *acsA*-encoding CODH catalytic subunit. This SNV caused an A97E amino acid change, and the mutant strain showed a 1.4-fold increase in both the growth rate and the CO consumption rate under autotrophic conditions of 44% CO syngas compared to the wild-type strain (Kang et al., 2020). These results show that C1 gas utilization efficiency can be increased by altering the kinetics of the CODH/ACS protein. Overexpression of such mutant CODH proteins may facilitate higher C1 gas utilization than wild-type CODH/ACS.

## Strategies to Overcome Energetic Limitations in C1 Gas Utilization

Acetogens suffer from insufficient energy supply in autotrophy, as they are known to live at the thermodynamic edge of life (Schuchmann and Müller, 2014). As mentioned earlier, the energy limitations of acetogens and the low solubility of gaseous substrates restrict the production of energetically high-cost metabolites. Fortunately, besides C1 gases, acetogens can metabolize diverse substrates, including sugars, alcohols, carboxylic acids, and methanol as alternative carbon or energy sources (Perez et al., 2013; Schuchmann and Müller, 2016). The metabolic flexibility of acetogens allows them to overcome energetic limitations by facilitating energy supply.

### Liquid C1 Feedstocks: Methanol and Formate

The electrochemical or photochemical reduction of CO_2_ generates formate and methanol, which are liquid forms of C1 feedstock with the benefits of mass transfer and energy efficiency (Cotton et al., 2020). Unlike C1 gases, they are easily soluble in water and are transportable, storable, and safe. The most promising aspect is their energy efficiency, as the conversion of methanol or formate is higher than that achieved with H_2_/CO_2_ or CO (Claassens et al., 2019).

**Figure 3:**
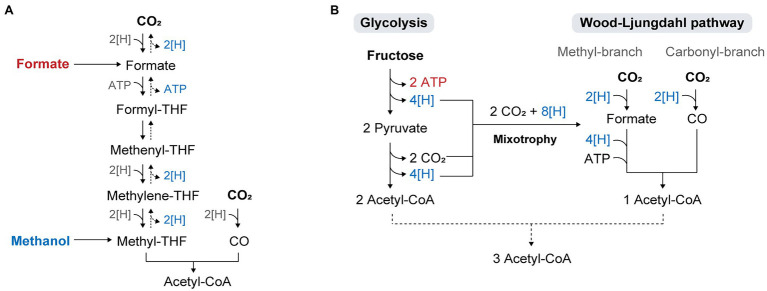
Metabolic flexibility of acetogens utilizing diverse substrates. **(A)** Direct assimilation of formate and methanol into the WL pathway. **(B)** Concurrent utilization of Glycolysis and the WL pathway in acetogenic mixotrophy.

Methanol and formate are directly assimilated into the WL pathway (Figure 3A). The utilization of these substrates in acetogens has been shown to improve cell growth and product yields of reduced chemicals such as butyrate. For example, *E. limosum* cultivated on methanol has not only shown higher growth rates than cells cultivated with C1 gases (Genthner and Bryant, 1987; Loubière et al., 1992), but also high product yields with the production of 12 mM acetate and 3.7 mM butyrate from 20 mM methanol (Litty and Müller, 2021). The yield of butyrate under methanol conditions was significantly higher than that obtained under H_2_/CO_2_ or CO conditions, where acetate was the major product while butyrate was produced in trace amounts. As methanol retains more reducing equivalents than gaseous substrates, a surplus of reducing equivalents can be used to drive biosynthesis and improve the conversion yield of butyrate. The increased butyrate-to-acetate ratio in the presence of methanol was assumed to be due to the role of butyrate production in the NAD(P)H-NAD(P)^+^ balance during methanol assimilation, as NAD(P)H generation from methanol is catalyzed by 3-hydroxybutyryl-CoA dehydrogenase to regenerate NAD(P)^+^ (Flaiz et al., 2021).

Similarly, *A. woodii* has been regarded as an interesting organism for anaerobic formate-based bio-production because of the high-energy efficiency achieved through acetate production from formate compared to that from H_2_/CO_2_ (Cotton et al., 2020). A recent study showed that the cultivation of *A. woodii* with formate as the sole carbon and energy source resulted in conversion of formate to acetate at a higher rate and efficiency than cultivation on gaseous substrates (Moon et al., 2021; Neuendorf et al., 2021). The study also compared the energetic efficiency of different acetogens and other common microbial hosts (e.g., formatotrophs or engineered strains capable of utilizing formate or methanol) during growth and product formation on C1 or sugar substrates, revealing that acetogens show superior energy efficiency on all substrates analyzed, with the highest values for C1 substrates. Formate showed an even higher energy potential than gaseous substrates. Among acetogens, *A. woodii* and *E. limosum* are promising biocatalysts for the conversion of formate into acetate, as they form acetate as a major product during growth on formate (Litty and Müller, 2021).

It should be noted that utilization of formate and methanol has several disadvantages. During formate consumption, pH of the medium can increase slightly (Litty and Müller, 2021; Moon et al., 2021), and the produced alcohols are toxic to some microorganisms at high concentrations, presumably because they damage the cell membrane and cause end-product inhibition of glycolytic enzymes (Dürre et al., 1988; Caldwell, 1989). The increase in pH during formate consumption can be controlled through addition of buffers during fermentation. Regarding the toxicity of methanol, adaptive evolution of acetogens in high concentrations of methanol can increase their tolerance to methanol, as shown by a previous study wherein an adapted strain of *S. ovata* achieved a 5-fold increase in the growth rate of methanol with a higher tolerance to the solvent (Tremblay et al., 2015). More importantly, the reduction of formate to acetate produces two molecules of CO_2_ in the absence of excess reducing equivalents to refix the released CO_2_, resulting in a carbon efficiency of only 50%. To reduce the carbon loss, addition of H_2_ can be a strategy to enable complete fixation of CO_2_, improve the carbon efficiency, and even facilitate net CO_2_ uptake (Molitor et al., 2016; Novak et al., 2021).

### Renewable Reducing Power: Electricity and Light

H_2_ and CO are poor electron donors compared to high-energy substrates, such as methanol and glucose, resulting in insufficient electron supply in acetogens (Emerson and Stephanopoulos, 2019). Instead, the direct supply of such electron sources could be a potential strategy to overcome cellular energy limitations in acetogens. Replacing H_2_ or CO with renewable reducing powers for gas fermentation has been demonstrated in acetogens with added advantages of the enhanced efficiency of C1 gas conversion and the metabolic shift toward reduced products (Nevin et al., 2011; Kracke et al., 2016; Im et al., 2018; Cheng et al., 2022).

Microbial electrosynthesis (MES) is a process in which microbes use electrons derived from an electrode to reduce CO_2_ to multi-carbon products (Nevin et al., 2010). Several acetogens have been reported to perform MES *via* extracellular electron transfer. For example, direct electron uptake from the electrode has been proposed for *S. ovata, Clostridium aceticum, C. ljungdahlii*, and *M. thermoacetica*, showing reduction of CO_2_ into organic acids; however, clear evidence of direct electron transfer is still lacking (Nevin et al., 2010, 2011). In contrast, *C. autoethanogenum* requires an electron mediator such as methyl viologen (MV) or neutral red to transfer electrons from the electrode to cells (Kracke et al., 2016). As some acetogens grow on the electrode by forming a biofilm (Nevin et al., 2010), electron transfer can be spatially limited to cells in contact with the electrode and affected by the biofilm density (Pirbadian et al., 2020), which can result in complex spatial patterns of activity in the bioelectrochemical system (BES).

Extracellular electron supply in the BES induced a significant metabolic shift in *C. autoethanogenum*, resulting in a significant reduction in acetate production, with increased production of lactate and 2,3-BDO by 35-fold and 3-fold, respectively, compared to that under conditions without the electron mediator (Kracke et al., 2016). Recently, *Clostridium carboxidivorans* was found to be capable of MES with MV or biochar as an electron mediator, which facilitated electricity-driven autotrophic CO_2_ fixation (Cheng et al., 2022). The standard redox potential of MV is −446 mV, which is sufficiently low to reduce ferredoxin (−450 to −500 mV) and NADH (−320 mV). Hence, it can regulate the redox balance and redistribute the redox flux in acetogens (Michaelis and Hill, 1933; Schuchmann and Müller, 2014). However, increasing the amount of MV supplementation has been reported to have toxic effects on cells (Cheng et al., 2022). Alternatively, neutral red is another electron shuttle with low toxicity and a standard redox potential similar to NADH (Harrington et al., 2015). Addition of neutral red was found to improve the production of volatile fatty acids from CO by increasing the reducing power (Im et al., 2018).

MES powered by solar energy enables artificial photosynthesis in acetogens, with the same net overall reaction as plant-based photosynthesis, in which CO_2_ and water are converted to organic compounds and oxygen (Nevin et al., 2010). Light-capturing catalysts, such as cadmium sulfide (CdS) or gold (Au) nanoparticles (NPs), are attached to cells and deliver electrons obtained from light to the cells. For example, *M. thermoacetica* displaying biologically synthesized CdS-NPs or Au-NPs were found to convert CO_2_ to acetate (Sakimoto et al., 2016; Zhang et al., 2018). However, the proposed system was limited by the biosynthesis of photo-responsive CdS, which decreases chemical production due to a defense mechanism against heavy metals in bacteria. To circumvent this issue, the chemically synthesized CdS-NP was attached to *C. autoethanogenum*, where the successful conversion of CO_2_ into acetate was demonstrated using only light-induced electrons from CdS-NPs (Jin et al., 2021). However, the mechanism of extracellular electron transfer from nanoparticles remains unclear. Transcriptional analysis of *C. autoethanogenum* attached to CdS-NPs suggested that electrons from CdS-NPs are transferred to cells indirectly through mediators such as iron or flavin mononucleotide (FMN). Another study conducted in *M. thermoacetica*-CdS suggested two types of electron transfer pathways: The H_2_ generation pathway mediated by membrane-bound hydrogenase and an H_2_-independent pathway (Kornienko et al., 2016). Interestingly, a recent study found that neither CdS-NPs nor light was involved in CO_2_ fixation, but cysteine present in the medium was used as the carbon source for acetate production in *M. thermoacetica* (Göbbels et al., 2021). These findings indicate that the underlying mechanism of this system is not fully understood; hence, more research is needed to exploit the potential of artificial photosynthesis systems with acetogens.

### Mixotrophy

Acetogenic metabolism operates at a thermodynamic limit, which results in an insufficient energy supply during autotrophy (Schuchmann and Müller, 2014). This problem becomes more critical during the cultivation of engineered acetogens, where antibiotics are added to maintain the plasmid, or when the engineered strains express heterologous biosynthesis pathways for energetically high-cost metabolites. In a study, limited autotrophic growth was observed in cells harboring an empty plasmid (Shin et al., 2019), and the non-native chemical mevalonate was only produced under fructose conditions because of the limited availability of reducing equivalents and ATP during C1 gas fermentation (Diner et al., 2018). To overcome the thermodynamic problem and improve autotrophic growth under such conditions, sugar co-feeding is a promising strategy. With a broad range of substrate utilization, acetogens can improve bioenergetics by using sugars along with C1 feedstocks in a process called mixotrophy, where carbon is fixed through both the glycolytic and WL pathways (Fast and Papoutsakis, 2012; Tracy et al., 2012; Fast et al., 2015). Sugar oxidation *via* glycolysis generates eight reducing equivalents and two moles of CO_2_, all of which are re-assimilated *via* the WL pathway (Figure 3B). Theoretically, it produces a total of three molecules of acetyl-CoA and hence, increases the yield of acetyl-CoA by 50% compared to standard Embden-Meyerhof-Parnas (EMP) glycolysis (Fast et al., 2015). However, in practice, some of the reducing equivalents are required for biomass generation and maintenance, which indicates that CO_2_ evolved in glycolysis cannot be fully converted to acetyl-CoA, resulting in carbon loss as CO_2_ and a decrease in the overall carbon yield. Hence, the choice of substrates co-fed with sugars is crucial for achieving complete carbon fixation and high product yields. Supplying exogenous reducing power such as H_2_ or syngas during sugar fermentation of acetogens has been demonstrated, which showed high productivity that exceeded the sum of individual substrate productivities and carbon efficiency over 90%, and promoted the generation of more reduced products (Jones et al., 2016; Maru et al., 2018; Cheng et al., 2019).

Another advantage of mixotrophy is the improvement in cellular growth due to the increased availability of acetyl-CoA obtained from additional substrates and improved cellular energy in acetogens. Glucose co-feeding has been shown to improve the methanol uptake rate in *E. limosum* with an increased growth rate and biomass yield due to the improved availability of both carbon and energy for anabolic reactions (Loubière et al., 1992). Recently, *A. woodii* showed mixotrophic growth on formate and fructose, where co-feeding of formate increased the acetate production rate by 50% compared to fructose utilization alone (Neuendorf et al., 2021). Formate utilization improved cell-specific acetate productivity, whereas fructose increased the overall bioenergetics, increasing the amount of ATP wasted. However, co-utilization of formate and fructose resulted in an equal production of CO_2_, which was released for growth solely on formate, with a carbon efficiency of only 50%. Although co-feeding of formate and fructose is not preferable to formate utilization alone in terms of carbon efficiency, addition of fructose could be used to improve the bioenergetics of formate-utilizing acetogens and facilitate the redirection of carbon flux from acetate toward desired products, such as ethanol and lactate, in metabolically engineered strains (Neuendorf et al., 2021). Further research is needed to improve carbon yield when formate is co-fed for mixotrophic cultivation.

Although mixotrophy is a promising strategy for increasing the bioenergetics of acetogens, a key concern in its implementation is the possibility of carbon catabolite repression (CCR) of the WL pathway in the presence of a preferred sugar substrate. CCR has been found to be dependent on species and culture conditions (Liu et al., 2015; Maru et al., 2018). For example, CCR in *C. aceticum* (Braun et al., 1981), *M. thermoacetica* (Huang et al., 2012), and *Blautia coccoides* GA-1 (Liu et al., 2015) inhibits autotrophic metabolism and causes poor H_2_/CO_2_ consumption when glucose or fructose is present at high concentrations. In contrast, *A. woodii, Butyribacterium methylotrophicum, C. autoethanogenum, C. carboxidivorans, C. ljungdahlii*, and *E. limosum* concurrently utilize sugars (glucose or fructose) and C1 gases without CCR (Jones et al., 2016; Maru et al., 2018). However, *E. limosum* was found to be affected by CCR when co-fed with methanol and glucose (Loubière et al., 1992).

Despite the promising aspects of mixotrophy, few studies have elucidated the CCR mechanism in acetogens or the interaction between the WL pathway and glycolysis. Therefore, a thorough understanding of mixotrophy is crucial to fully exploit its potential without CCR. Nevertheless, researchers have recently discovered solutions to overcome CCR in mixotrophy. Controlling the glucose feeding rate with kinetically limiting concentrations during the continuous fermentation of *M. thermoacetica* successfully prevented CCR (Park et al., 2019). This shifted the carbon substrate preferences toward CO_2_ and helped achieve mixotrophic growth without CCR. Another recent study similarly employed xylose-limited conditions, in which the simultaneous uptake of xylose and CO for acetate production was observed in *C. autoethanogenum* (Mann et al., 2020).

## Biotechnological Applications of Acetogens

With the enhanced performance of the WL pathway and bioenergetics achieved through the aforementioned strategies, acetogens can increase the yields and titers of native metabolites such as acetate, butyrate, and ethanol (Bertsch and Müller, 2015a; Katsyv and Müller, 2020). In recent years, several efforts have been made to improve the product selectivity of native metabolites by modifying culture conditions (Table 2).

**Table 2:** Strategies to improve product selectivity of native or non-native biochemicals in acetogens.

Target product	Species	Product type^*^	Strategy	References
Ethanol	*C. autoethanogenum*	Native	Deletion of *adhE1a* increased ethanol production to 2.46 g/L on CO fermentation	Liew et al., 2017
	*C. aceticum*	Native	Medium acidification increased ethanol production to 4.4 g/L from CO	Arslan et al., 2021
	*C. carboxidivorans*	Native	Medium acidification stimulated conversion of acids into alcohols during syngas fermentation	Fernandez-Naveira et al., 2017
Isopropanol	*C. ljungdahlii*	Non-native	Reinforcing acetate reassimilation by overexpressing *aor* and acyl-CoA synthetases (*acs* and *fadKM1/M2*) reduced acetate byproduct and enhanced production of isopropanol and ethanol on syngas fermentation	Jia et al., 2021
3-HB	*C. ljungdahlii*	Non-native	Downregulation of *pta via* CRISPRi increased 3-HB production by reducing acetate production	Woolston et al., 2018
Butyrate	*E. limosum*	Native	Addition of acetate in gas fermentation increased butyrate production with a shift of major product from acetate to butyrate	Loubière and Lindley, 1991; Park et al., 2017
	*E. limosum*	Native	Feeding methanol promoted butyrate production	Flaiz et al., 2021; Litty and Müller, 2021
	*B. methylotrophicum*	Native	Varying methanol-to-bicarbonate ratios in the culture media affected butyrate yield and selectivity	Wang et al., 2021
	*C. ljungdahlii*	Non-native	Deletion of *pta, adhE1* and CoA transferase homolog increased butyrate synthesis to 1.3 g/L, reducing acetate production under H_2_/CO_2_ fermentation	Ueki et al., 2014
	*C. ljungdahlii*	Non-native	Downregulation of *adhE1 via* CRISPRi increased butyrate production by reducing ethanol synthesis	Zhao et al., 2019
Butanol	*E. limosum*	Native	High methanol-to-formate ratios induced butanol production with a titer of 38 mg/L	Wood et al., 2021

* *Non-native products indicate that the corresponding biosynthesis pathway is introduced into the acetogen to produce the target chemical*.

### Culture Conditions to Shift Metabolite Profiles

#### Increasing Alcohol Selectivity

Metabolite formation by acetogens is dependent on the culture conditions, including gas composition, substrate, and pH (Arslan et al., 2021; Bae et al., 2022). Most importantly, the availability of a sufficient amount of reducing equivalents is a key factor, as it constrains metabolic flux distribution, energy management, and product formation in acetogens (Hermann et al., 2020). CO was found to be the preferred substrate over H_2_/CO_2_, glucose, or fructose in *C. ljungdahlii, C. carboxidivorans*, and *C. aceticum* for the production of alcohols (Fernandez-Naveira et al., 2017; Hermann et al., 2020; Arslan et al., 2021). The reduction of CO in the carbonyl-branch generates Fd_red_, which is then oxidized *via* Rnf to provide NADH. The increased supply of both Fd_red_ and NADH during CO fermentation can facilitate alcohol production by activating aldehyde:ferredoxin oxidoreductase (AOR) or promoting the reaction of bifunctional aldehyde/alcohol dehydrogenase (Hermann et al., 2020; Liu et al., 2020; Arslan et al., 2021). Several studies have found that AOR plays a significant role in ethanol production during autotrophic growth in CO-containing syngas cultures (Richter et al., 2016; Valgepea et al., 2017, 2018). Similarly, medium acidification by pH drop stimulated ethanol production in *C. aceticum* grown on CO, whereas the same effect was not observed for fructose fermentation (Arslan et al., 2021). Similarly, the medium acidification stimulated *C. carboxidivorans* to convert acids to alcohols when grown in a syngas mixture, but this effect was not observed during glucose fermentation (Fernandez-Naveira et al., 2017).

Interestingly, modifying the co-substrate ratios can induce the production of unexpected metabolites because of the improved bioenergetics in acetogens. Recently, varied ratios of methanol-to-formate were tested in *E. limosum*, where a methanol-to-formate substrate ratio of 7.5:1 achieved a maximum butanol titer of 2.0 ± 1.1 mM (38 mg/L). This is the first evidence of native butanol production in *E. limosum*, as it has not been observed under only syngas or methanol fermentation (Wood et al., 2021). Although the underlying mechanism and butanol production pathway in *E. limosum* remains elusive, butanol production is suspected to be due to overflow metabolism, similar to 2,3-BDO production in *C. ljungdahlii* or *C. autoethanogenum* (Köpke et al., 2011).

#### Increasing Butyrate Selectivity

*E. limosum* was reported to produce butyrate only during CO-fed syngas fermentation, and not when using H_2_/CO_2_, as in the latter case, most of the carbon is directed to acetate synthesis for ATP generation (Litty and Müller, 2021). Butyrate production in acetogens not only provides the ATP needed for cell growth but also balances the redox, indicating that butyrate yield and selectivity depend on NADH availability (Flaiz et al., 2021; Wang et al., 2021). The production of butyrate has been reported to be enhanced by the supplementation of additional reducing equivalents or methanol to provide more NADH (Flaiz et al., 2021; Fu et al., 2021). In addition, the amount of bicarbonate added to the medium can affect the redox levels. When bicarbonate is present in excess in the medium, CO_2_ reduction consumes more NADH, resulting in lower NADH availability for reassimilating acetate to produce butyrate. This effect was confirmed in *B. methylotrophicum*, where supplementation with 20 mM bicarbonate and 100 mM methanol significantly improved butyrate production, making it the major product (Wang et al., 2021). In contrast, 40 mM and 60 mM bicarbonate induced the accumulation of a large amount of acetate. Acetate is another co-substrate that influences butyrate selectivity. The addition of acetate into the culture medium during gas fermentation of *E. limosum* increased butyrate production compared to pure gas fermentation (Loubière and Lindley, 1991; Park et al., 2017). Supplementation with acetate elevated energy status and shifted the major product from acetate to butyrate.

### Strain Engineering for Non-native Biochemical Production

The development of genetic tools and synthetic biology approaches has opened up possibilities for the metabolic engineering of several acetogens (Köpke et al., 2010; Shin et al., 2019; Zhao et al., 2019; Jin et al., 2020). Using such tools, the heterologous expression of biosynthetic pathways for producing desired chemicals has expanded the product spectrum of acetogens, including isoprene, isopropanol, acetone, and 3-hydroxybutyrate (3-HB; Jones et al., 2016; Diner et al., 2018; Woolston et al., 2018; Karim et al., 2020; Jia et al., 2021). Although they were proof-of-concept production, as their titers were considerably low due to concurrent production of native metabolites as byproducts, significant improvements can be achieved with strain engineering that redirects carbon fluxes toward desired chemicals.

Blocking competing pathways is an effective method for redirecting the carbon flux. Deletion of three genes encoding PTA, AdhE1, and CoA transferase in *C. ljungdahlii* increased butyrate synthesis and reduced acetate and ethanol byproducts (Ueki et al., 2014). CRISPR-mediated downregulation of *adhE1* also redirected the carbon from ethanol to butyrate in engineered butyrate-producing *C. ljungdahlii* (Zhao et al., 2019).

Considering that acetate synthesis is coupled with ATP formation and is essential for energy supply in acetogens living in energy-limited autotrophic conditions, blocking acetate-producing pathways can result in poor cell growth (Huang et al., 2016). A feasible strategy to overcome this issue is to reassimilate the acetate to form the target product. The AOR present in acetogens converts acetate into ethanol with acetaldehyde as an intermediate. Its role in acetate reassimilation has been found in a study in which knockout of AOR enzymes significantly impaired the conversion of acetate into alcohols in *C. autoethanogenum* (Liew et al., 2017). The positive effect of this strategy was confirmed by the overexpression of native AOR in *C. carboxidivorans,* which resulted in higher ethanol production (Cheng et al., 2019). Recently, a novel acetate reassimilation pathway containing two acyl-CoA synthetases (ACS from *C. ljungdahlii* and FadKM1/2 from *E. coli*) that catalyze acetate conversion to form acetyl-CoA was tested in *C. ljungdahlii* along with overexpression of native AOR enzymes (Jia et al., 2021). Introduction of the pathway into an engineered strain to produce isopropanol successfully reinforced acetate assimilation, achieving significantly reduced acetate formation and increased production of ethanol and isopropanol through syngas fermentation.

## Conclusion and Future Perspectives

Acetogens are attractive microorganisms that can convert C1 gases into value-added biochemicals. Recently, as carbon-neutral technologies have received much attention to mitigate climate change, acetogens have been considered as promising biocatalysts capable of fixing C1 gases. To date, many studies on acetogens have been conducted to understand the enzymes involved in the WL pathway and to convert acetyl-CoA into value-added chemicals. As various omics analyses based on systems biology approaches have been applied for studying acetogens, systems-level understanding of metabolic pathways, including the WL pathway and energy conservation system, has accumulated and expanded our knowledge on acetogenic metabolism. An example is the transcriptional or translational regulation of genes encoding enzymes of the WL pathway, based on the transcriptome or translatome analyses (Marcellin et al., 2016; Al-Bassam et al., 2018; Kremp et al., 2018; Shin et al., 2018, 2021; Song et al., 2018; Neuendorf et al., 2021). In addition, several genome-scale metabolic models have been constructed for several acetogens. They have provided information to develop a chassis strain and tools for *in silico* simulations, such as intracellular carbon flow or cellular energy prediction in acetogenic metabolism (Liu et al., 2019; Mahamkali et al., 2020; Song et al., 2020; Zhu et al., 2020). Recently, protein structure prediction tools, such as AlphaFold2 or RoseTTAFold, have been launched (Baek et al., 2021; Jumper et al., 2021). They are expected to play an important role in revealing the functions and mutations of many proteins in the WL pathway and energy conservation systems. Furthermore, proteins involved in the WL pathway may be engineered or a novel C1 fixation pathway could be designed and constructed using deep learning tools. In addition, genome manipulation techniques, whose application in studying acetogens has been limited, are also being rapidly developed. In particular, the genome-editing technologies based on CRISPR/Cas have been applied in engineering acetogens to identify genes essential for cell growth and metabolite production (Huang et al., 2016; Shin et al., 2019; Zhao et al., 2019; Jeong et al., 2020; Xia et al., 2020). Furthermore, the development of various genetic parts, modules, and circuits based on the synthetic biology approach is expected to enable the transcriptional or translational regulation of genes involved in the acetogenesis under specific culture conditions. Therefore, we expect that the application of various studies based on systems and synthetic biology approaches to acetogen engineering will further improve the C1 gas conversion efficiency and ultimately lead to the development of highly efficient biocatalysts for C1 gas fixation.

## Author Contributions

B-KC conceptualized and supervised the project. HL, JB, SJ, SK, and B-KC wrote the manuscript. All authors contributed to the article and approved the submitted version.

## Funding

This work was supported by the C1 Gas Refinery Program (2018M3D3A1A01055733 to B-KC) through the National Research Foundation of Korea (NRF) funded by the Ministry of Science and ICT (MSIT).

## Conflict of Interest

The authors declare that the research was conducted in the absence of any commercial or financial relationships that could be construed as a potential conflict of interest.

## Publisher’s Note

All claims expressed in this article are solely those of the authors and do not necessarily represent those of their affiliated organizations, or those of the publisher, the editors and the reviewers. Any product that may be evaluated in this article, or claim that may be made by its manufacturer, is not guaranteed or endorsed by the publisher.
